# Supplementary material for: Seminoma and Embryonal Carcinoma Footprints Identified by Analysis of Integrated Genome-Wide Epigenetic and Expression Profiles of Germ Cell Cancer Cell Lines
Source: PLoS One. 2014 Jun 2;9(6):e98330. doi: 10.1371/journal.pone.0098330 (PMC4041891; doi:10.1371/journal.pone.0098330)
Supplement: File S1 — ZIP file containing DMRforPairs output for significant regions. Please start from the html files. (ZIP) [file pone.0098330.s008.zip › figures/42166.pdf]

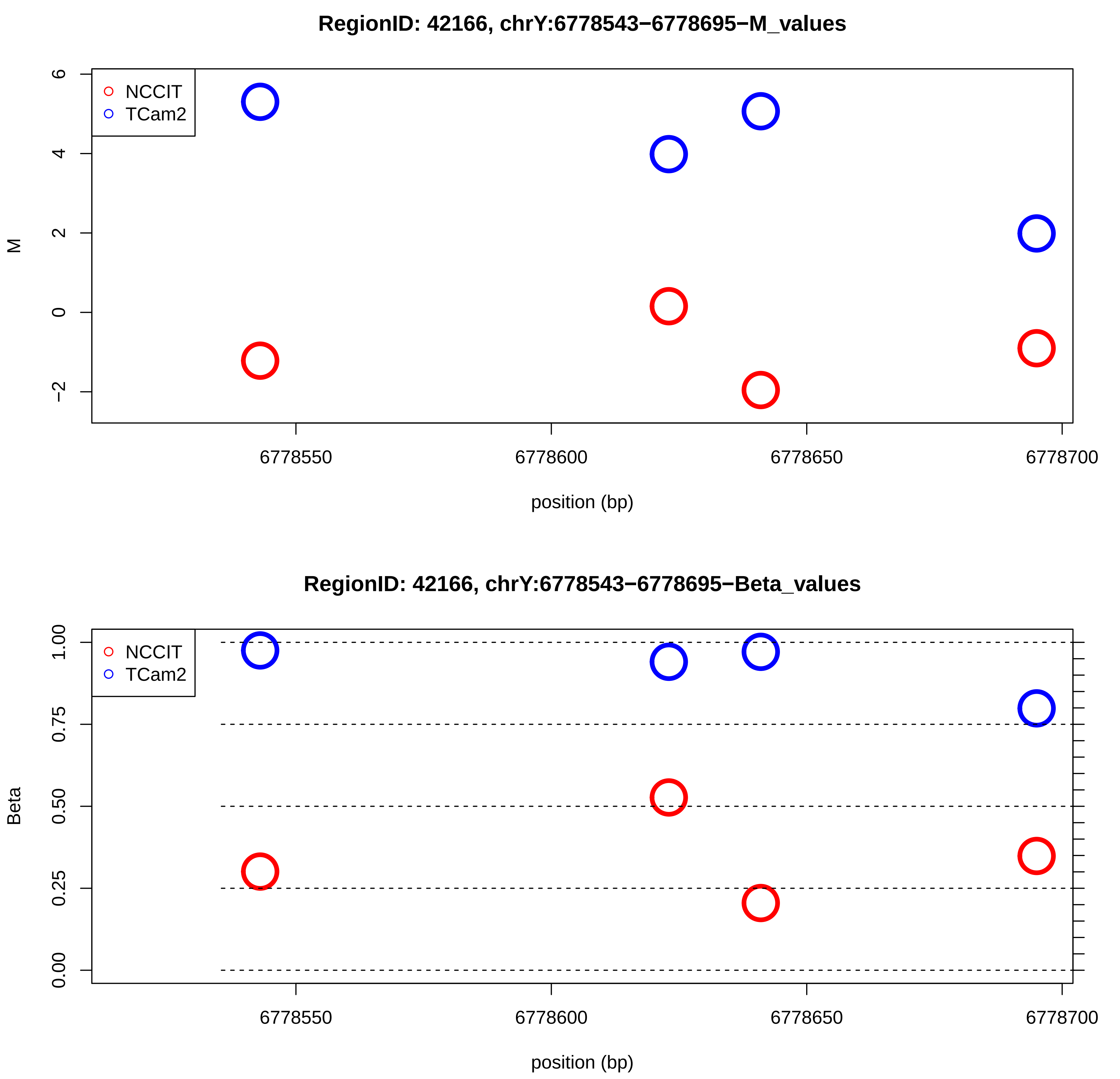
RegionID: 42166, chrY:6778543−6778695−M_values
NCCIT
TCam2
M
6
4
2
0
−2
6778550
6778600
6778650
6778700
position (bp)
RegionID: 42166, chrY:6778543−6778695−Beta_values
NCCIT
TCam2
Beta
1.00
0.75
0.50
0.25
0.00
6778550
6778600
6778650
6778700
position (bp)
